# The role of law in the control of obesity in England: looking at the contribution of law to a healthy food culture

**DOI:** 10.1186/1743-8462-5-21

**Published:** 2008-10-14

**Authors:** Robyn Martin

**Affiliations:** 1 Centre for Research in Primary and Community Care, University of Hertfordshire, UK; 2 The Chinese University of Hong Kong, Shatin, NT, Hong Kong; 3 London School of Hygiene and Tropical Medicine, Keppel Street, London, UK

## Abstract

Obesity levels in England are significantly higher than in much of the rest of Europe. This article examines aspects of the physical and cultural context of food consumption in England, and the evolution of government policy on obesity, as a background to an analysis of how law might play a role in obesity prevention. Research suggests that individual food choices are associated with cultural and socio-economic circumstances and that they can be manipulated by advertising, food packaging and presentation. This suggests that there might be ways of using law to manage the influences on food choices, and of using law in support of strategies to redirect food choices towards healthy food products. Law is a particularly useful tool in the protection of the individual against the economic power of the food industry, and there is much that law can do to change the physical, economic and social environment of food consumption.

## Background

While obesity levels in Europe are generally lower than those in the United States and Australia [1], the prevalence of obesity is increasing. Obesity rates vary widely across Europe, ranging from low levels of obesity in Switzerland to significant levels in Spain and the United Kingdom [2]. The substantial variation across European states with similar economic profiles suggests that physical, cultural and social environments might be contributing factors to obesity. We are beginning to understand the relationship between the physical environment and obesity [3], enabling law to intervene to regulate the built environment so as to provide healthier cities. In Europe, as in North America [4] and Australia [5], it is now becoming increasingly clear that socio-demographic and behavioural factors are also significant risk indicators for obesity. Research demonstrates that individual food choices are associated with socio-economic circumstances and food cultures [6]. Food choices can be negatively manipulated by advertising, food packaging and presentation of food content [7]. This suggests that choice might also be manipulated to achieve positive health outcomes. Levels of physical activity, for example, can be influenced by factors such as the ways in which physical education is offered [8,9]. Law has the power to influence behaviour, and the intervention of law may serve to enable healthier food choices and change health behaviours.

It is now widely accepted, across the western world at least, that law has a role to play in controlling obesity by changing the environments in which food is consumed [10]. Law has the power to control commercial food practices, to regulate physical and economic environments, to regulate media practices, and to support informed consent by requiring the provision of information. Law also serves to influence public attitudes to eating behaviours and to create expectations of health standards. The World Health Organisation (WHO) has not yet used its treaty making powers for obesity along the lines of the 2003 Framework Convention on Tobacco Control, but it has initiated a Global Strategy on Diet, Physical Activity and Health [11] to support policies that promote accessibility to foods low in fat, salt and sugar [12]. WHO has since published further more detailed reports [13] for the purpose of developing an evidence base on mechanisms for reducing obesity levels. The reports recognise that self regulation has not proved sufficient, particularly in reducing the volume of food and drink marketing to children or in minimizing the effects of marketing. Self regulation operates as a third alternative to the more intrusive government regulation and to no regulation at all, and usually consists of a voluntary association of organizations with the objective of controlling their collective action [14]. WHO recommends that in the context of food marketing, self regulation operate in a legal framework in which there are incentives for compliance, especially where children's health is at risk [15].

This article will examine the context of obesity in England, looking at factors which might have relevance for the culture of food choices and health behaviours contributing to weight gain, and for the interventions which might address those factors. The paper will then examine ways in which law is used for obesity prevention, ways in which law might be used, and arguments that have been raised both for and against the use of law as a public health tool in the context of obesity in England.

## The law and obesity context – 10 things you need to know about England

Obesity is one of the most pressing public health concerns in England. Public health interventions to prevent increasing levels of obesity are a government priority [16]. 22% of men and 23% of women in England are classified as obese, and the Department of Health predicts that by 2010, this will rise to 33% of men and 28% of women [17]. Obesity has become a public obsession. Newspapers, popular magazines, television and radio programmes and government sound bites have created a considerable body of information and advice, often conflicting, about how to lose weight. The language is at times sensational and emotive. Newspapers have proclaimed an 'Obesity time bomb', a 'crisis with devastating implications for the nation's health' [18], and a 'toxic time bomb' where children are 'doomed to be overweight' [19]. The language can also be judgemental. Obesity is 'largely a consequence of people eating junk food and leading a sedentary lifestyle' [20]. To be obese in contemporary England is to be an object of public scrutiny and increasingly, an object of public condemnation on the assumption that obesity is an issue of self-control. Yet public health research suggests that 'uncertainty over the aetiology of obesity remains one of the chief barriers to designing effective strategies for prevention and treatment' [21].

Each and every country has its own food and health culture. It is important to identify influences and constraints that will affect both the culture of obesity and the practice of public health in England if we are to attempt to design legal solutions for the English obesity problem. Solutions that work elsewhere may not be appropriate if transported wholesale without reference to local culture. These are some of the issues we will need to take into account.

### 1. The United Kingdom is a federal system

The four countries of the United Kingdom, England, Wales, Scotland and Northern Ireland, have different histories, different cultures, different health and legal systems, and different government powers. Health is a delegated government responsibility, so legislation pertinent to health is generally unique to each UK country. Yet many activities and services, such as the media, are provided nationally across the UK and there is free movement across borders. This creates problems for legal interventions for the benefit of public health, as state measures may be confounded by national interventions.

### 2. The United Kingdom is a member of the European Union

Governments within the United Kingdom are not always free to make their own laws. EU states are bound to implement European Directives. While the EU has not been particularly active in the field of public health as such, it has been active in prescribing regulation of the content and source of food products, and has legislated on issues such as the physical environment and the free movement of goods and health personnel within Europe, all of which might have implications for obesity [22]. EU regulation has worked both for and against public health. The food and environmental quality in England may have improved, but the European Common Agricultural Policy (CAP) has been credited with increasing the price of healthy food by its subsidies for the withdrawal and destruction of good quality fruit and vegetables to maintain prices [23,24]. Similarly, national legislation regulating television content in any state in the European Union is weakened by the European *Television without Frontiers *Directive, which specifies that broadcasting is governed by the laws of the country in which it originates. This would allow English viewers to access television programs from other European states where there is no regulation of food advertising.

### 3. The health system in the United Kingdom is a National Health System

This means that for persons choosing to use NHS healthcare services, all health care is free at the point of delivery. The state, and hence the taxpayer, bears the full economic burden of obesity-related disease, possibly at the expense of other healthcare treatments. The potential cost of chronic disease to the health system has provided considerable impetus for government action on obesity. The cost element of obesity plays a greater part in the obesity debate in the United Kingdom than in states with private healthcare systems, on the assumption that individual obesity causes economic harm to the state. These cost factors can distort public health initiatives. It has been argued, for example, that persons who have become obese as a result of their lifestyle choices should be denied NHS services. Assisted fertility specialists have proposed that childless women who are obese should not be eligible for NHS fertility treatment [25]. Arguments against regulation based on the right to individual lifestyle choices are considered to have less weight when it is the state, and consequently other taxpayers, who are required to pay for adverse consequences of choice.

### 4. The class system in English culture and its implications for health inequalities

England is now much more egalitarian than was historically the case, but despite refashioning of the categorization of differentials between individuals to avoid classifications on the basis of class, there remain considerable disparities in socio-economic status [26]. Obesity levels reflect these disparities. This is more than an issue of people with money and people without. The area where people live will have consequences for availability of fresh fruit and vegetables, opportunities for sport and exercise, access to medical care and health advice, quality of schooling, and street safety to enable walking to work, school and shops. There are also entrenched divisions in food preferences, and typically the main dietary differences lie in the nutritional content of foods [27]. The relationship between obesity and socio-economic status is complex. In England, evidence suggests that those from lower socio-economic backgrounds have a greater risk of obesity [28]. Elsewhere, in Asian states such as Hong Kong for example, prevalence of obesity is more likely to be associated with affluence than poverty [29,30], and in Belgium highly educated men, who are less likely to undertake military service, are more likely to be obese [31].

### 5. Long working hours culture

People in England work longer hours than anywhere else in Europe. Long working hours are perceived as demonstrating commitment to work, and peer pressure can mean that those who choose to go home at the end of the normal working day are regarded as not taking the job seriously. There may also be the need to improve take-home pay by doing overtime to keep up with the consumer culture. Working hours are often compounded by long commutes to and from work. Long hours mean that parents may not be home to eat with their families, and that there is less time for food preparation. Changing work patterns mean that families are now much more likely to eat snack and convenience foods containing increased levels of fat, salt and sugar [32]. The significance of long working hours for obesity has been recognised in the House of Commons Select Committee Report on Health [33].

### 6. Children do not eat with their parents

It has long been the case in England that children generally eat their evening meal separately from their parents. For the moneyed classes, children have traditionally eaten tea early in the nursery, and then at boarding school [34]. For poorer families, the availability of convenience foods and long working hours have increasingly meant that children, tired from the school day, do not wait up to eat with their parents later in the evening. As Warde has noted, this has contributed to the re-ordering of the time-space relations in every day life [35]. In England there has developed the concept of 'children's food'. In most other cultures, young children graduate from a diet of baby food to family food. In England, there is a genre of food (fish fingers, baked beans, chicken nuggets) which is designed and marketed especially for children.

This has had two consequences. The first is that because this is children's food, children have more control over what is served. Children do not have to compromise with the food tastes of adults, and they can demand the food that they like. Secondly, children have become a market in their own right for manufacturers pushing easily prepared foods of poor nutritional value [36,37]. This is a valuable market, and manufacturers are reluctant to lose it.

The English Medical Association argues the importance of establishing a healthy eating pattern in early life [38], when children acquire many of the physical attributes and social and psychological structures for life. Family meal patterns have been found to have relevance for obesity levels [39,40]. Obesity measures will need to target the child food culture as well as the adult food culture.

### 7. Children eat school dinners

Most English school children eat their lunches in the school canteen. This could provide an opportunity to ensure that children have a proper healthy meal, and certainly this was the original intention of school dinners. In 1906 a legal framework was introduced for the provision of school food, and regulations required school meals to meet prescribed nutritional standards [41], but these standards were abolished in 1981 as a component of the dismantling of the welfare state [42].

A recent enquiry suggested that some schools spend as little 37p per day on a child's meal, resulting in the provision of cheap, processed food [43]. Private schools spend more than twice as much on school meals as state schools [44]. State school catering arrangements allow children to eat snack foods and foods high in fat, salt and sugar, and much of the food served in school canteens is fast food [45]. National concern about the quality of school meals has been led by the 'Naked Chef' Jamie Oliver and his *Feed me Better *campaign, forcing the government to address the issue of school food.

### 8. England has low rates of breastfeeding

Research demonstrates a link between breastfeeding and prevention of childhood obesity [46,47]. Breastfeeding rates in the United Kingdom are among the lowest in Europe and lower than in many other states [48]. In the United Kingdom 76% of mothers breastfeed their babies on discharge from hospital [49] but that figure drops to 42% by the time the baby is 6 weeks old. In Australia for example, 84% of mothers initiate breastfeeding, and they continue to breastfeed for longer [50].

### 9. English climate

English winters are cold, and winter days are short. Although there is insufficient evidence on the relationship between cold climate and obesity [51], there is evidence to suggest that seasonal affective disorder and related depression, more common in northern climates [52], is relevant to binge eating [53,54]. In winter the traditional English diet includes comfort foods – hot and filling foods such as fish and chips and sticky toffee pudding, sweet foods such as chocolate with high sugar content, and snack foods such as crisps containing high levels of fat. There is a tendency in England to hibernate through the winter and to watch more television, and there is an association between long television hours and obesity [55].

### 10. Binge drinking

Alcohol consumption by young people in England has increased dramatically in recent years, especially among women. The consumption of alcohol in England is higher than anywhere else in Europe [56]. Binge drinking has been shown to be associated with obesity [57]. While government policy has addressed problems caused by alcohol, there has been little attention given to alcohol as a cause of obesity. The government's Alcohol Strategy makes no reference to the fact that most alcoholic drinks have a calorific value as high as a high-sugar soft drink. However the House of Commons Select Committee Report on Obesity in 2004 recommended that the Department of Health commission research into the correlation between trends in alcohol consumption and obesity [58,59].

These are just some aspects of the context of obesity in England. There are others, such as the monopoly of major supermarket chains on food retailing, agricultural policies with the potential to distort the pricing of fresh foodstuffs, the crowded physical environment of English cities and traffic policies which favour vehicle over pedestrian and bicycle use. All of these issues warrant further examination, and law has a possible role in the regulation of all these policies and activities in the furtherance of public health protection.

## Government policy on obesity in England

Former Prime Minister Tony Blair's Labour government issued a series of papers on health, and it is worth tracking changing government policy approaches to obesity. We can begin with the National Audit Office paper, *Tackling Obesity in England*, in 2001 [60,61]. This paper served not so much to provide solutions, but rather to highlight the problems caused by obesity, and to identify who within the National Health Service was responsible for dealing with those problems. The paper set out 'to make recommendations that might help to create a climate in which individuals are aware of the consequences of obesity, and can make informed decisions about their lifestyle' [62]. The paper recommended a number of NHS initiatives such as strategies to help people to lose weight, and the preparation of national guidelines on obesity for health authorities. The thrust of this paper was that obesity was a ***medical ***problem, and as such the responsibility for obesity lay with the NHS and its healthcare services.

This was followed by the Wanless Reports in 2002 [62] and 2004 [63-65]. Derek Wanless was given a brief by the Treasury to determine the long term resource requirements of the National Health Service. The outcome of the Wanless reports was that obesity became essentially an ***economic ***problem. Health problems resulting from obesity were identified by Wanless as likely to put significant strain on future NHS budgets.

The House of Commons Select Committee Report on Obesity was published in 2004. The brief here was to identify both the health implications of obesity and the social and economic costs, so as to make recommendations for government strategy. At this point obesity appears to have become a ***social ***problem:

The causes of obesity are diverse, complex, and in the main, underpinned by what are now entrenched societal norms [66].

and,

...we believe that the most important and dramatic changes will have to take place outside the doctor's surgery, in the wider environment in which people live their lives...the main factors contributing to the rapid rise in obesity are societal...it is critical that obesity is tackled first and foremost at societal rather than individual level [67].

In the long awaited public health white paper, *Choosing Health: Making healthy choices easier *[67], published shortly after the Select Committee Report, obesity was identified as one of six government health priorities. Given the dire economic predictions in the Wanless reports, the approach taken in *Choosing Health *was not surprising. The title of the paper made clear the thrust of government policy – *choosing *health. The paper explained that in earlier times, when the greatest threat to health was communicable disease, the role of government in the prevention of ill-health was top-down. State responsibility for health reflected the cultural and political relationship between citizens and state at that time. But times had changed, and in an era of threat from non-communicable disease we needed a new approach to public health which recognised freedom of individual choice in a diverse, open and more questioning society. In this context, obesity became a ***public health ***problem:

Health is inexplicably linked to the way people live their lives and the opportunities available to choose health in the communities where they live [68].

The categorisation of obesity as a public health issue is significant. The state, through the National Health Service, is responsible for providing medical care services, so if obesity is a medical problem then obesity is a state responsibility. But if obesity is a public health issue, then there is shared responsibility between the state and the population. Public health policy was now directed to helping people choose better lifestyles. The *Choosing Health *paper proposed a range of initiatives to create greater health opportunities with promises of better health education (such as the 5 A DAY fruit and vegetable initiative), better health advice (the appointment of 'NHS health trainers' and 'Personal Health Care Kits') and the preparation of an obesity prevention strategy to give guidance to individuals on how to change their lifestyles. More focused *Choosing Health *papers followed. The '*Choosing Health? Choosing a better diet*' consultation paper [69], for example, proposed strategies for improving food supply, improving food information and improving nutrition in schools and in the NHS. Underpinning all these strategies was the philosophy of shared responsibility:

We must recognise, however, that individuals also have to take responsibility for their diets and the diets of people in their charge. [70]

The role of government is to:

...support consumers, providing them with easier access to a wide range of healthier foods and, crucially, the information and knowledge needed to make informed choices about their diets. And this may mean targeting action to meet the needs of particular groups and tackle inequalities. [71]

From this position of shared responsibility, subsequent policy documents have gone on to develop a stronger 'personal responsibility' for health theme. Tony Blair said July 2006 [72]:

Our problems are not, strictly speaking, public health questions at all. They are questions of individual lifestyle.... They are not epidemics in the epidemiological sense. They are the result of millions of individual decisions.

Thus obesity became, finally, a ***personal ***problem. The former Prime Minister suggested that what people now wanted was a government that saw its role as empowering the individual, not trying to make choices for him. This, he said, can only work on the basis of a different relationship between citizen and state. The government's role was to provide practical support for people who lacked the basic skills so as to help them to use health information. The Minister for Public Health agreed, '...we are talking about some very simple messages – take a bit more exercise, eat better, make sure your children do the same' [73]. The Health Secretary noted that individuals needed now to take responsibility for their own health, 'We've already stepped in but there's only so much the government can do...People need to want to change their lifestyles and take responsibility for their health' [74].

There has, however, been criticism of government policy on obesity, in particular that a focus on personal responsibility leads to oversimplification of the problem. Evidence shows that the causes of obesity are complex:

...it has been multiple small changes in society which have contributed to the changing population weights. ...we are going to have to intervene in multiple ways to push it back down again, there is not one simple answer [75].

## The role of law in the control of obesity in England

The thrust of government policy is important for the way in which law might play a role in obesity control. If, for example, obesity is a medical problem then responsibility for reducing obesity levels will lie with the organisation of medical services, and the role of law will be limited. If obesity is an economic or social problem, then law can work to alter the economic or social environment. If obesity is a public health problem, then law can authorise public health interventions into private life in the same way that law works to enable interventions for the control of communicable disease. If, however, government policy is to pass responsibility to the individual, then what is the role of public health law in the control of obesity? In government policy papers there has been little reference to law as a public health tool. Policy predicated on individual responsibility and choice does not leave much room for regulation and compulsion in its armoury.

Yet the choices we are being asked by the government to make are not made in a vacuum. Can we rely on food manufacturers to give us accurate and accessible information on food content to enable us to make informed food choices? Can we rely on food marketers to be honest in their media advertising campaigns? Can we choose to ride our bicycles to work where there are no bicycle paths and the traffic is dangerous? If the child's environment is crucial to patterns of obesity, how realistic is it to argue that the child in a hostile health environment must take responsibility for choosing health? A coordinated government policy together with a well researched obesity strategy is a good starting point, but law has tools at its disposal that can ensure that policy is effective.

In particular law can:

• impose enforceable duties on bodies which are in a position to improve the health environment

• provide powers (such as powers of licensing, taxation, inspection) which give some leverage in ensuring that stakeholders recognise their responsibilities

• provide tools such as judicial review and actions in tort to enable private bodies and individuals to protect health

• provide protections against public health interventions which go too far and which impinge on the human rights of individuals

• set norms to influence public opinion on what is and what is not acceptable health behaviour

So in the context of the English health environment as discussed above, how might law help?

## Law to reduce long working hours

The European Union Working Time Directive [76] became law in the United Kingdom in October 1998. The Directive sets a maximum working week of 48 hours, but allows in exceptional circumstances for employers to agree with employees to extend working hours. The former conservative Thatcher government refused to implement the Working Time Directive, but this refusal was successfully challenged in the European Court of Human Rights [77]. The Directive was then implemented in the United Kingdom, but subject to the right of workers to agree to opt out of the maximum working week. As a result of the UK opt-out provisions, it is common for workers to be expected to work long hours. Over a quarter of full time employees working in the United Kingdom work in excess of 48 hours a week [78]. Long working hours have implications for food preparation and family eating patterns, which in turn have consequences for childhood obesity [79]. The House of Lords has defended the opt-out [80], arguing that English employers need flexibility to ensure global competitiveness, using rights arguments such as that the opt-out 'preserves the right of those who want or need to work overtime'.

A change in the law to meet the spirit as well as the letter of the EU directive would have significant benefits for health, especially for food preparation and family eating practices, but also for family life. The French statutory Employment Code, for example, limits the working week to 35 hours, with a recent amendment allowing employees to work up to 39 hours, at extra cost to the employer, English industry argues that this has been a disaster for the French economy [81] and would be harmful for England [82]. Not all commentary agrees [83], and there is evidence that work is now organised more effectively and productively as a result of the Directive. What is not in dispute is the better diet in France (French families are more than twice as likely as English families to eat food prepared from base ingredients [84]), and the better management in France of the work/life balance [85]. It is a question of priorities. If the government is serious about obesity, then a change in the law on working hours to support a change in working culture, such that family time is recognised and valued, would be a starting point.

## Law to support breastfeeding

The European Commission document *Protection, promotion and support of breastfeeding in Europe: a blueprint for action *[86] recommends the use of national legislation to support breastfeeding. Legislation can work to discourage formula feeding, to protect maternity leave, to enforce the provision of safe breastfeeding environments in industry for working women, and to remove obstacles and barriers to breastfeeding in public places. English law currently prevents baby milk products from being prescribed for supply by pharmaceutical services [87] and prohibits the advertising of milk products for infants under 6 months of age [88], but does not regulate 'follow on' milk formula products for babies over 6 months or the use of baby milk logos in health literature and elsewhere. Organisations such as UNICEF, The National Childbirth Trust and Save the Children UK are calling for stronger legal protection for new mothers against pressure to feed their babies infant formula [89].

Women in England who breastfeed in public areas have been threatened with public indecency offences on the argument that their behaviour is offensive to members of the public. Scotland passed legislation [90] creating an offence to prevent a woman from breastfeeding in a shop, restaurant or any public place. A private member's bill for similar laws was introduced in England with the support of the National Childbirth Trust, but there was considerable opposition on grounds that this was a 'nanny state' intervention. In 2007 a range of government departments prepared a consultation paper addressing residual inequalities not covered by existing discrimination legislation [91]. The recommendations included increasing protection against discrimination in the provision of goods, services and premises on the grounds of pregnancy and maternity, and may well be interpreted to cover the case of the manager of a restaurant or café asking a breastfeeding woman to leave the premises. The government has now proposed that in its forthcoming Equality Bill, women be protected in relation to the breastfeeding in public of babies up to six months old. Maternity campaigners are urging the government to recognise the legal right to breastfeed in public for babies of all ages. European Union guidelines recommend that employers make provision for breastfeeding at work, and that mothers who are breastfeeding be given flexible working hours, but these guidelines have not been given statutory force in the UK. Again, changes in the law that have as their specific objective the protection of the rights of women to breastfeed, and the provision of premises and services designed to enable breastfeeding, could assist in the obesity strategy.

## Law to regulate school food

In 2000, concerned about the poor quality of school meals, the government passed regulations introducing nutritional standards for school lunches. The regulations required that some fruit and vegetables be offered as part of every school meal [92], but did not prevent the serving of fast food. It was left up to the child to choose between the healthy food and other food offered.

The government has now accepted the recommendation of the School Meals Panel Report that guidelines, advice and voluntary compliance by schools do not work to reduce the fast food intake of children [93]. Child choice of food does not necessarily result in healthier eating. Policy needs the support of law.

New nutrition regulations have now been passed for school meals served in primary schools, mandating what food can be offered to children and the nutritional content of school food [94]. Every child must be given at least two portions of fruit and vegetables per day, and there must be regular servings of fish and meat. These requirements have now been extended to the content of school vending machines [95], although it is recognised that this will result in considerable losses in funding to schools [96].

## Law to regulate food labeling

The Food Safety Act 1990 makes it an offence to sell food that is injurious to health. The expression 'injurious to health' has a legal meaning, and requires proof of causation between the food complained of and the harm. This can be established in the case of immediate harms such as food poisoning, but will be overwhelmingly burdensome in the case of long term harms. It would not be easy to establish that the hamburger you ate in 2006 was the cause of your coronary heart disease ten years later. Food safety legislation deals with the safety element of food and not the nutritional content, and so provides no remedy for obesity.

In his 'Healthy Living' speech, Tony Blair acknowledged that people can only choose health if they are given accurate information on which to base their choices. Food labeling is governed in England by the Food Labeling Regulations 1996, in accordance with European Union law. Information on the nutritional content of food products is required only when a nutritional claim is made by the manufacturers. Food content is required to be labelled in a prescribed form, but that form has not been addressed to the lay purchaser, and is not always easy to interpret. Claims of the fat content in food are often misleading, and many low fat foods do not mention high sugar content. Campaigners have called for new food labeling laws which would enable consumers to choose healthy foods without having to undertake mathematical calculations at the point of purchase. There have been many proposals on the presentation of information on food content including 'traffic light' systems for healthy foods, points systems, 'treat food' symbols and signposting.

Food manufacturers and retailers, a powerful economic force, have opposed attempts to label food as healthy or unhealthy, fearing discrimination against snack foods such as confectionery. They point out that there is no such thing as bad food (unless it is injurious to health in the sense covered by the Food Standards Act), and that comparisons with tobacco regulation are inappropriate. As one manufacturer said about a chocolate bar:

...health warnings are for dangerous things. Whilst we recognise the problem I do not think that a Curly Wurly is a dangerous thing. [97]

This view has had government support [98] but it is now accepted that some foods can be identified and classified as healthy or unhealthy. The food industry itself has begun to promote what it call a 'healthy eating' range of foods.

The Food Standards Agency accepts the need to review the law to make nutrition labeling compulsory and to indicate levels of fat content. Law is necessary because under a voluntary scheme, there would be no guarantee that all manufacturers and retailers would indicate content in the same way. The EU is also looking at revising its legislation on nutrition labeling to make it compulsory [99]. English government still favours a voluntary approach [100]:

We know that busy consumers want a single clear system to help them inform themselves about the food they are buying in the shops. We are encouraging the food industry to adopt the Food Standard Agency's clear system for food labeling...It will be much better if the industry comes together voluntarily around this scheme but once again, we are prepared to act if the voluntary system does not work.

But as Lang has noted,

The role of law in the governance of the relationship between food and public health is being altered by the changed structures and dynamics of modern food systems...public health is being stretched by a new set of dynamics in which perfectly legal actions by food marketers...have a sometimes unwitting impact on public health [101].

The range of forms of food labeling across the main processed food providers in England continues to result in confusion in consumer understanding of nutrition content. For example Waitrose, Sainsbury and the Co-op supermarkets use the Food Standards Agency recommended 'Traffic Light' system, but the UK's largest supermarket, TESCO, has opted for a 'Signposting' system of labeling. While there is as yet limited evidence that food labeling will result in a reduction in levels of obesity, statutory presentation of food content that is common across all prepared food, in a form that makes clear the content and meaning of nutritional components of food, would assist in informed, healthy food choices.

## Law to control food advertising to children

The most hotly contested area of legal intervention in obesity control in the United Kingdom is the proposal to prohibit the advertising of unhealthy foods that is targeted at children. The foods most commonly advertised to children in the United Kingdom [102], as elsewhere [103], are those that have high levels of fat, salt and sugar.

The role of advertising in children's food choices is now well recognised, and health campaigners have called on the government to use law to regulate food advertising to children [104]. The House of Commons Health Committee notes '...the food industry's relentless targeting of children through intense advertising and promotion campaigns, some of which explicitly aim to circumvent parental control by exploiting "pester power"' [105]. The Food Standard Agency has accepted the '...causal link between promotional activity and children's food knowledge, preference and behaviours' [106]. Government policy has begun to recognise that voluntary guidelines and choice are not always the answer. As former Prime Minister Tony Blair stated:

where children are concerned, I have come to the conclusion that we need to be tougher, more active in setting standards and enforcing them [107].

The Government proposed [108] that "if there was not a change in the nature and balance of food promotion by early 2007, (it) would take action to implement a clearly defined framework for regulating the promotion of food to children". The Office of Communications (Ofcom) [109] was asked to consider proposals for strengthening its rules on television advertising of food to children. In March 2006 Ofcom published a consultation document in which it concluded that self-regulation alone would not be sufficient to deal with the problem of advertising to children, and that there was a case for strengthening the rules on advertising to reduce the exposure of young children to unhealthy food products. The document proposed three alternative solutions:

• timing restrictions on HFSS (high in fat, salt and sugar as measured by means of the Food Standards Agency guidance on nutrient profiling) food and drink products targeted at children under 10 years old

• timing restrictions on all food and drink products targeted at children under 10 years old

• volume based restrictions on all food and drink products at different times of the day

Ofcom's brief was not to consider regulation but rather to make recommendations for a voluntary code of practice within the food industry on the basis that 'if by 2007 the voluntary code hasn't worked, we will make it mandatory' [110].

There was a strong response from the food industry, which argued that restrictions based on nutrient profiling are wrong in principle as a regulatory tool. Few foods are harmful in moderation and regulation would demonise some foods as unhealthy [111]. At the same time, health campaigners were almost universally critical of the consultation document, particularly Ofcom's rejection of the proposal that there be a complete ban on advertising HFSS products before the 9 pm watershed [112]. Similarly, there was criticism of the stated objective of the OfCom enquiry, which was to develop measures that balanced health and social benefits against the costs to broadcasters if food advertising were restricted. The Royal Society of Public Health for example argued that:

The avoidance of cost to broadcasters should not be "balanced" against the public interest of children's health. The public health objective (and the purpose of this code review) should be the overriding priority [113].

In response to these concerns Ofcom issued a further consultation document in December 2006 [114]. Again there was a strong response from food advertisers. The Food Advertising Unit, established under the auspices of the Advertising Association, argued that advertising restrictions will have little or no impact on childhood obesity, and that food advertising had 'at best only a "modest" direct impact on children's food preferences' [115]. Ofcom published its final conclusions and recommendations in February 2007 [116]. From April 2007 advertisements for HFSS products were prohibited in or around programmes made for children, or in and around programmes likely to be of particular appeal to children, aged 4 to 9 years of age. In January 2008, this was extended to programmes likely to be of interest to children between 4 and 15 years of age. From 2009 HFSS foods will be banned from all children's channels.

It is recognised that food marketing to children goes beyond media broadcasting to encompass sponsorship, promotions, product placement and internet marketing [117], and that legal regulation of television advertising will provide only a partial solution to marketing of unhealthy foodstuffs. Ofcom's remit is limited to radio and television advertising, and since the broadcasting prohibitions came into force many food and drink manufacturers have transferred their attention to advertising on internet sites of interest to children, such as the social networking site Bebo [118]. In March 2007 the Department of Health initiated a further exploration of food advertising on non-broadcast sites such as the press, posters, cinema, the internet, labels, wrappers and packaging, as well as sponsorship and brandsharing [119]. The Advertising Standards Authority code of conduct has been extended to cover 'new media' including internet advertising, but this does not extend to advertising which can be classified as an 'editorial' on a brand's own website. Children can be invited to play a computer game on a brand website and then be exposed to advertising. There is much still to be done to protect children from manufacturers' attempts to influence food preferences.

Meanwhile, national government initiatives may be overtaken by EU developments on the *Television Without Frontiers *Directive which aim to amend regulations on the protection of minors, applicable to all audiovisual services in Europe [120].

## Conclusion

The allocation of responsibility to individuals for overweight and obesity in England is becoming increasingly entrenched. Recent initiatives include treating childhood obesity as evidence of childhood neglect so that social service legal powers can be brought into play to remove children from parental environments and to prosecute parents [121], the classification of some over-eating behaviours such as Prader-Willi Syndrome as evidence of mental illness for the purposes of detention powers under the Mental Health Act [122], and blaming obesity for rising levels of cancer [123]. The focus of government health policy in the United Kingdom on responsibility and choice rather than on the public health context of obesity has mitigated against the use of law and regulation for the furtherance of public health goals. Yet even in this environment of personal responsibility for health, law can still be a useful tool for instigating change to the physical and socio-economic environments [124] that influence the way we live and the lifestyle choices we make.

Law has had some success in other public health contexts such as alcohol abuse [125] and tobacco consumption [126], but we must be careful in assuming that the same solutions will apply to foodstuffs. The distinctions between good and bad, healthy and unhealthy foods are complicated to legislate for. The media environment has changed since alcohol and tobacco measures were first introduced and it is now the case that media advertising has a more global [127] and diverse dimension. The role of law in dealing with obesity must go far wider than the regulation of food.

There is much that can be done to ensure that the conditions in which people make food and food pattern choices facilitate a full, free and informed choice. Government policy on obesity has not addressed many of the factors which contribute to lifestyle choices, factors where the benefits may be more measurable and less politically difficult than in relation to food control. There is much that can be done now which is not being done, and law has not been usefully and effectively exploited in English obesity strategy. Framing legal solutions may not be easy, and they may not be watertight. But law is not only about enforcement. As noted by former Prime Minister Tony Blair, 'Legislation can, itself, help to change a culture. To outlaw an activity is ...a strong signal that...behaviour is unacceptable' [128]. The power of law to alter health behaviours, to create expectations of health standards, and to change our collective ways of thinking about threats to public health has yet to be harnessed in efforts to control obesity in England. If law is to play a role in protection against obesity and its associated health harms, we must continue build an evidence base to determine the contributions of factors such as geography, climate, economics, media, patterns of food trade and distribution and culture to obesity. Only then can we be confident that law can be framed effectively to support obesity strategies.

## Competing interests

The author declares that he has no competing interests.
